# New Evidence, Creative Insights, and Strategic Solutions: Advancing the Understanding and Practice of Diabetes Osteoporosis

**DOI:** 10.1111/1753-0407.70091

**Published:** 2025-04-23

**Authors:** Bei Tao, Ximei Shen, Guangfei Li, Xiyu Wu, Yuying Yang, Chunxiang Sheng, Yun Zhang, Ling Wang, Zhiyun Zhao, Qi Song, Dewen Yan, Sunjie Yan, Youjia Xu, Huijuan Yuan, Houde Zhou, Jianmin Liu

**Affiliations:** ^1^ Department of Endocrine and Metabolic Diseases, Shanghai Institute of Endocrine and Metabolic Diseases Ruijin Hospital, Shanghai Jiao Tong University School of Medicine Shanghai China; ^2^ Shanghai National Clinical Research Center for Metabolic Diseases, Key Laboratory for Endocrine and Metabolic Diseases of the National Health Commission of the PR China, Shanghai Key Laboratory for Endocrine Tumor, State Key Laboratory of Medical Genomics, Ruijin Hospital Shanghai Jiao Tong University School of Medicine Shanghai China; ^3^ Department of Endocrinology, The First Affiliated Hospital Fujian Medical University Fuzhou China; ^4^ Department of Orthopedics, Osteoporosis Clinical Center The Second Affiliated Hospital of Soochow University Suzhou China; ^5^ National Clinical Research Center for Metabolic Diseases, Key Laboratory of Diabetes Immunology, Ministry of Education, and Department of Metabolism and Endocrinology The Second Xiangya Hospital of Central South University Changsha China; ^6^ Department of Endocrinology, Henan Provincial Key Medicine Laboratory of Intestinal Microecology and Diabetes, Henan Provincial People's Hospital People's Hospital of Zhengzhou University Zhengzhou China; ^7^ Department of Radiology, Beijing Jishuitan Hospital, Capital Medical University National Center for Orthopedics Beijing China; ^8^ JST Sarcopenia Research Centre, Beijing Research Institute of Traumatology and Orthopedics, Beijing Jishuitan Hospital Capital Medical University, National Center for Orthopedics Beijing China; ^9^ Department of Radiology, Ruijin Hospital Shanghai Jiao Tong University School of Medicine Shanghai China; ^10^ Department of Endocrinology, Shenzhen Second People's Hospital, the First Affiliated Hospital of Shenzhen University, Health Science Center of Shenzhen University Shenzhen Clinical Research Center for Metabolic Diseases Shenzhen China

**Keywords:** diabetes, diagnosis, fractures, osteoporosis

## Abstract

**Background:**

Diabetes osteoporosis is a debilitating condition that significantly impacts human health. However, it is often underdiagnosed and not addressed in a timely or appropriate manner.

**Methods:**

Recent studies were reviewed to explore the roles of energy metabolism, sarcopeina, low‐grade inflammation and gut microbiota in the development of diabetes osteoporosis.

**Results:**

Osteoporosis in diabetic patients differs from primary osteoporosis. Novel biomarkers and risk factors that are biologically, physiologically, and pathologically linked to the development of diabetes osteoporosis are emerging, necessitating a shift in strategies for diagnosis, risk stratification, and prevention of diabetes osteoporosis.

**Conclusions:**

There is an urgent need to approach this disorder from a fresh perspective, initiating a range of basic research and clinical investigations.

## Graphical abstract

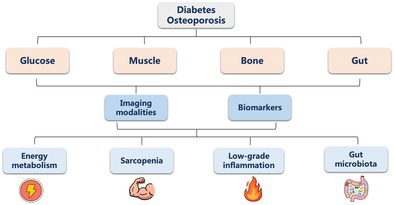

**Summary**

- The outcome of diabetes osteoporosis is more than an elevated fracture risk; it is associated with higher cardiovascular disease risk and all‐cause mortality.
- Diabetes osteoporosis is not a disease simply with the combination of diabetes and osteoporosis.
- The complex mechanism of diabetes osteoporosis includes, but is not limited to systemic energy metabolism, insulin resistance, sarcopenia, chronic low‐grade inflammation, gut microbiota, and etc.
- Novel imaging modalities to detect the microstructure damage of bone in diabetes are needed.

Diabetes is one of the most prevalent chronic diseases worldwide, and its chronic complications, including cardiovascular, renal, retinal, and neurological damage, pose serious threats to human health and result in significant social and economic burdens. In addition to these traditional complications, the impact of diabetes osteoporosis is gaining increasing attention. The risk of fragility fractures in patients with type 1 diabetes (T1D) and type 2 diabetes (T2D) is reportedly 20%–50% greater than that in nondiabetic individuals [1]. More concerning is the nearly twofold increase in cardiovascular disease risk in diabetic patients with a previous fracture compared with those without a fracture history [2], and the mortality risk for those with osteoporotic vertebral fractures is 110% and 82% greater than that for those with diabetes alone or fractures alone [3]. Therefore, the prevention and treatment of diabetes osteoporosis are highly important.

In recent years, research on diabetes osteoporosis has led to new evidence that osteoporosis in diabetic patients differs from primary osteoporosis, necessitating a shift in strategies for its diagnosis, risk stratification, and prevention.

## 1 Characteristics of Diabetes Osteoporosis

### 1.1 Fracture Risk in Diabetes Patients Cannot Be Fully Captured by Existing Diagnostic Methods

The diagnosis of osteoporosis is dependent mainly on dual‐energy X‐ray absorptiometry (DXA). However, compared with nondiabetic individuals, diabetic patients may not exhibit lower bone mineral densities (BMDs) and may even have higher BMDs. This “diabetic bone paradox” is observed not only in individuals with T2D but also in individuals with T1D under 20 years of age. The fracture risk in diabetic patients is even independent of BMDs, suggesting that, to some extent, diabetes itself is a condition with equivalent fracture risk.

The 10‐year osteoporosis fracture risk assessment tool FRAX is widely recommended for fracture risk assessment and for guiding the initiation of osteoporosis treatment. However, this tool underestimates fracture risk in diabetic patients. A 10‐year follow‐up study in 1700 T2D patients revealed that the FRAX‐calculated 10‐year risk of major osteoporotic fractures in diabetes‐corrected models was 52%–62% lower than the actual incidence [4].

These clinical challenges highlight the limitations of using traditional DXA as the “gold standard” for diagnosing osteoporosis or FRAX for assessing fracture risk in diabetic patients. These findings underscore the necessity and importance of establishing new diagnostic systems and fracture risk stratification standards for diabetes osteoporosis.

### 1.2 Diabetes Osteoporosis Is Neither an Age‐Related Disease Nor a Chronic Complication of Diabetes

Primary osteoporosis is commonly observed in postmenopausal women and elderly individuals, but applying this approach to diabetes patients may lead to significant underdiagnosis. Epidemiological data show that the fracture risk in T1D patients begins to exceed that of nondiabetic individuals by 10% starting at age 30 [5], and the fracture risk in prediabetic patients is 2.77‐fold greater than that in those with normal glucose tolerance; however, in those over 40 years of age, the risk increases by 57% [6]. These results suggest that the target population for diabetes osteoporosis prevention should include prediabetic patients.

Chronic diabetic complications such as nephropathy [7] and neuropathy [4] are associated with osteoporosis and fracture risk. However, in a 10‐year follow‐up study in T2D patients in China, HbA1c and disease duration were not associated with fracture risk [4]. A multicenter, open‐label, randomized, parallel study in Japan involving 2542 T2D patients revealed no difference in cumulative fracture incidence between groups with traditional therapy (targeting HbA1c, blood pressure, and LDL‐C less than 6.9%, 130/80 mmHg and 120 mg/dL, respectively) and intensified treatment (HbA1c < 6.2%, blood pressure < 120/75 mmHg, and LDL‐C < 80 mg/dL), after an average follow‐up of 7.8 years [8]. These findings suggest that diabetes osteoporosis is neither an age‐related disease nor a chronic complication of diabetes.

### 1.3 Unclear Temporal Relationship Between Diabetes and Osteoporosis

Currently, most of the understanding of diabetes osteoporosis stems from studies in diabetic patients. However, bone plays an essential regulatory role in glucose and energy metabolism. For example, osteoblast‐derived lipocalin2 and osteocalcin can modulate insulin secretion, insulin resistance, and appetite [9]. In a study of healthy nondiabetic individuals, the baseline bone resorption marker was not only positively correlated with HbA1c but also predicted impaired glucose metabolism after 4 years [10]. It was thus hypothesized that the human body sacrifices bone (leading to osteoporosis) to prevent diabetes [10]. Clinical studies have shown that administering anti‐bone resorption drugs, such as bisphosphonates or denosumab, to nondiabetic individuals reduces the incidence of diabetes, possibly due to lower serum dipeptidyl peptidase‐4 levels and higher glucagon‐like peptide‐1 levels after denosumab treatment [11, 12]. These findings support the idea that osteoporosis may precede diabetes, with bone having a role in predicting, preventing, and treating diabetes [10].

## 2 Key Factors to Consider in Studying the Pathogenesis of Diabetes Osteoporosis

### 2.1 Energy Metabolism

Diabetes is a disorder of energy metabolism, as is the case for diabetes osteoporosis. Basic studies have shown that knockout or overexpression of the insulin receptor in osteoblasts alters systemic insulin resistance and bone metabolism. In diabetic patients, the insulin resistance index is negatively related to bone strength and positively related to BMD.

The mechanism underlying the biological nature of diabetes osteoporosis as a disease of energy metabolism is complicated. Osteoblasts utilize aerobic glycolysis as an energy source, and its suppression in osteoblasts leads to compromised bone strength [13]. On the other hand, impaired glycolysis and oxidative phosphorylation in bone marrow mesenchymal cells contribute to impaired bone formation in diabetic mice [14].

Obesity, as commonly observed in individuals with diabetes, is often accompanied by fat accumulation and an increased number of senescent cells in the bone marrow, resulting in the differentiation of bone marrow mesenchymal stem cells toward adipogenesis rather than osteoblastogenesis. Bone marrow adipocytes promote osteoclast activity by increasing reactive oxygen species (ROS) production and secreting cytokines such as IL‐6. These changes are directly associated with decreased bone mechanical performance and an increased fracture risk [15].

At the molecular level, several signaling pathways in the skeleton, adipose tissues, and hypothalamus, such as the Wnt/β‐catenin [16], OPG/RANK/RANKL [17], leptin‐serotonin‐sympathetic nervous system [18], and VitD‐VDR signaling pathways, and genes, including fat mass and obesity‐associated genes (FTOs) [19] and secretin [20], are involved in the cross‐talk between bone and energy metabolism.

These findings support the idea that exploring the pathogenesis and prevention of diabetes osteoporosis through systemic energy metabolism is rational and necessary.

### 2.2 Sarcopenia

Sarcopenia is biologically linked to osteoporosis and other frailty states and can even predict mortality risk [21]. Compared with controls, T2D patients have higher rates of sarcopenia and fractures and lower bone quality, as assessed by the trabecular bone score (TBS) [22]. The impaired skeletal muscle regeneration in diabetic patients is also closely related to obesity and insulin resistance [23]. In addition, muscle density is an independent risk factor for subsequent hip fractures and mortality [24]. Thus, it is highly important to include sarcopenia in the etiology and risk assessment of diabetes osteoporosis.

### 2.3 Chronic Low‐Grade Inflammation

The development of diabetes, osteoporosis, and sarcopenia is associated with systemic chronic low‐grade inflammation, and immune dysfunction is a key mechanism underlying diabetic osteoporosis. In a hyperglycemic environment, abnormal osteoblast glycolysis and mitochondrial autophagy [13, 25] promote increased ROS production, which activates various proinflammatory signaling pathways, including MAPK, STAT1, STAT6, and NF‐κB, leading to M1 macrophage polarization and subsequent osteoclast differentiation. In hyperglycemia, the number of proinflammatory CD4^+^ T cells, which promote bone resorption, increases, whereas the number of Treg cells, which have bone‐protective effects, decreases [26]. Bioinformatics analysis revealed that high glucose levels activate the TNF‐α/FAS/FASLG signaling pathway, creating a chronic inflammatory microenvironment that promotes osteocyte apoptosis and inhibits osteoblast differentiation [27]. High glucose also enhances muscle cell inflammation and stimulates downstream pyroptotic signaling pathways, leading to reduced muscle cell numbers and impaired function [28].

Toll‐like receptor 4 (TLR4) is a transmembrane protein that plays crucial roles in initiating both innate and adaptive immune responses. It is a key molecule in classic inflammatory pathways and serves as a “hub factor” linking glucolipotoxicity with bone metabolism disorders. Knockout of TLR4 improves glucose and lipid metabolism in diabetic rats, alleviates metabolic inflammation, promotes osteogenesis, inhibits osteoclastogenesis, and effectively mitigates the occurrence of diabetes osteoporosis. Further mechanistic studies confirmed that TLR4 mediates glucolipotoxicity‐induced osteogenic dysfunction through multiple mechanisms, including regulating the nuclear translocation of inflammatory factors, the activity of the Ca2^+^‐binding protein family, autophagy, and ferroptosis, ultimately inhibiting osteoblast differentiation [29].

### 2.4 The Gut Microbiota

The gut microbiota can regulate whole‐body metabolism, is associated with obesity, metabolic syndrome, diabetes, and several age‐related conditions, such as frailty, sarcopenia, and cognitive decline, and is involved in the pathogenesis of osteoporosis.

Diabetic patients often present with gut microbiota dysbiosis, with a reduced abundance of *Prevotella* species, increased *Lactobacillus* abundance, and decreased bacterial diversity. This dysbiosis influences bone homeostasis through increased inflammation and impaired gut barrier function [30] and affects the dynamic balance between bone and muscle health, which is considered a biological basis for sarcopenia. For example, short‐chain fatty acids (SCFAs), such as butyrate, regulate muscle satellite cell homeostasis and function, positively impacting skeletal muscle health [31].

Targeting the gut microbiota has emerged as a novel intervention for diabetes osteoporosis. A review of 45 studies on probiotic supplementation in healthy individuals revealed that noninvasive interventions involving probiotic supplementation could improve the abundance of specific bacteria in the gut microbiota, thereby positively influencing bone health. The consumption of fiber‐rich foods (e.g., whole grains, legumes, vegetables, and fruits) can increase gut microbiota diversity and promote SCFA production. Regular physical activity improves the gut microbiota composition and SCFA production, benefiting bone and muscle health. Moreover, prebiotics (e.g., inulin and fructooligosaccharides) and probiotics (e.g., Bifidobacterium and Lactobacillus) can increase beneficial bacterial populations. Fecal microbiota transplantation has been shown to improve diabetic neuropathy [32], which is an independent risk factor for diabetes‐related fractures [4]. While the underlying mechanisms linking the microbiota, diabetic neuropathy, and fractures remain unclear, these findings suggest new research directions on whether microbiota interventions can prevent and treat osteoporosis by improving chronic complications in individuals with diabetes.

### 2.5 Biomarkers

Many reviews have detailed the roles of various serum biomarkers in reflecting bone turnover and fracture risk in diabetic patients. Low bone turnover may contribute to increased fracture risk in diabetic patients. The formation of advanced glycation end products (AGEs) is a major mechanism responsible for various chronic diabetes complications. AGEs can accumulate in bone collagen fibers, making them stiffer, disrupting collagen mineralization, and compromising bone strength. This has become a significant mechanism for increased bone fragility in diabetes [33]. Pentosidine, a product of AGE accumulation, is associated with vertebral fractures in T2D patients and is independently negatively correlated with the TBS. In prospective observational studies and *meta*‐analyses, urinary pentosidine levels were found to be independently correlated with fracture risk in community‐dwelling elderly individuals [34] and to predict vertebral fractures in T2D patients [35]. Fluorescence measurements of AGE levels in the skin can predict the risk of coronary heart disease and diabetes and are associated with fracture risk independent of age, sex, and BMD [36].

While these serum and urine biomarkers are linked to BMDs and fracture risk in diabetic and nondiabetic patients, future research requires in‐depth analysis of bone tissue from fractured patients with and without diabetes to identify novel biomarkers specifically related to fractures in diabetic patients. These biomarkers could help predict fracture risk more precisely than traditional biomarkers in diabetic individuals.

### 2.6 Noninvasive In Vivo Assessment of Bone Microstructure via Novel Imaging Methods

In recent years, the use of imaging techniques for chronic disease screening has become a trend. As a disease characterized by impaired bone quality, early and accurate assessment of bone microstructure in diabetes patients is crucial for reducing fracture risk. Noninvasive imaging methods, including the DXA‐derived trabecular bone score (TBS), quantitative computed tomography, and peripheral quantitative computed tomography, can capture bone quality defects in patients with diabetes better than DXA. A lower TBS is frequently observed in individuals with diabetes/prediabetes than in controls and is associated with greater fracture risk, longer disease duration, and higher HbA1c in individuals with T2D [37, 38]. The thresholds of the TBS for diagnosing diabetes osteoporosis should be investigated and verified.

Furthermore, high‐resolution imaging techniques are needed, alongside low radiation doses, to assess bone quality in vertebrae and hips for diabetes osteoporosis. In this context, ultrahigh‐resolution photon CT technology, an emerging CT imaging method, significantly reduces radiation doses compared with traditional CT and achieves a spatial resolution of 0.2 mm. It can clearly visualize small vessels, arterial walls, distal bronchi, and trabecular bone [39]. This technology has shown remarkable success in the early identification of cardiovascular changes and precise quantification of fat content in the liver [40]. This novel imaging modality could be introduced for in vivo assessment of bone microstructure in diabetic patients.

## 3 Solution Strategy

Currently, the diagnoses and treatments for diabetes osteoporosis face challenges, including inaccurate diagnoses, the absence of early warning systems, and imprecise interventions, which focus only on elderly diabetic patients and those with chronic complications. Identifying novel biomarkers and risk factors that are biologically, physiologically, and pathologically linked to the development of diabetes osteoporosis is crucial.

Specifically, we must break old paradigms and establish new ones, focusing on three key “breaks” and three corresponding “establishments”: breaking the traditional reliance on BMDs for diagnosing diabetes osteoporosis and establishing new diagnostic standards and fracture risk stratification systems; breaking the limitations of neglecting the pathophysiological essence of diabetes osteoporosis and establishing novel specific biomarkers and intervention targets; breaking the narrow focus on elderly individuals and those with chronic diabetes complications; and establishing prevention and treatment strategies starting from high‐risk diabetic populations.

Methodologically, we can focus on systemic energy metabolism, insulin resistance, sarcopenia, chronic low‐grade inflammation, and the gut microbiota of diabetic patients to identify novel biomarkers. Advanced imaging techniques should be used to clearly differentiate and measure changes in bone microstructure in diabetic patients, enabling early fracture risk assessment.

The use of existing diabetes management populations and diabetes osteoporosis research cohorts to conduct collaborative studies in four key areas—glucose, muscle, bone, and microbiota—exploring the mechanisms and risk factors for diabetes osteoporosis is recommended. A structured megadata platform that includes clinical data, imaging genomics, inflammatory genomics, and metagenomics should be established. The use of machine learning and artificial intelligence algorithms in disease prevention and diagnosis should be fully leveraged. Through continuous exploration, testing, and optimization, we aim to simplify complex processes and ultimately create a new, integrated diagnostic standard and fracture risk stratification system for diabetes osteoporosis that aligns with the biological essence of the disease and is widely applicable.

Research on diabetes osteoporosis will continue to deepen our understanding, enhance practical capabilities, and overcome bottlenecks, with the goal of advancing the diagnosis and treatment of diabetes osteoporosis to new heights. Our objective is “Make Sweet Bone Strong.”

## Author Contributions

Bei Tao, Ximei Shen, Guangfei Li, Xiyu Wu, Yuying Yang, Chunxiang Sheng, Yun Zhang, Ling Wang, Zhiyun Zhao, and Qi Song participated in the literature search, analysis, and summary. Dewen Yan, Sunjie Yan, Youjia Xu, Huijuan Yuan, Houde Zhou, and Jianmin Liu clarified the writing framework, suggested implementation strategies, and modified the manuscript. Jianmin Liu was responsible for providing overarching guidance, proposing directional searching and writing strategies, and finalizing revisions, reviews, and approving the manuscript for submission.

## Conflicts of Interest

Jianmin Liu is an associate editor/Editorial Board member of *Journal of Diabetes* and a co‐author of this article. To minimize bias, he was excluded from all editorial decision‐making related to the acceptance of this article for publication. The other authors have no conflicts of interest to declare.
